# Individualised variable-interval risk-based screening for sight-threatening diabetic retinopathy: the Liverpool Risk Calculation Engine

**DOI:** 10.1007/s00125-017-4386-0

**Published:** 2017-08-24

**Authors:** Antonio Eleuteri, Anthony C. Fisher, Deborah M. Broadbent, Marta García-Fiñana, Christopher P. Cheyne, Amu Wang, Irene M. Stratton, Mark Gabbay, Daniel Seddon, Simon P. Harding, for the Individualised Screening for Diabetic Retinopathy (ISDR) Study Group

**Affiliations:** 1 Department of Medical Physics and Clinical Engineering, Royal Liverpool University Hospital, Liverpool, UK; 2 Department of Eye and Vision Science, Institute of Ageing and Chronic Disease, University of Liverpool, William Henry Duncan Building, 6, West Derby Street, Liverpool, L7 8TX UK; 3 St Paul’s Eye Unit, Royal Liverpool University Hospital, Liverpool, UK; 4 Department of Biostatistics, Institute of Translational Medicine, University of Liverpool, Liverpool, UK; 5 Gloucestershire Retinal Research Group, Cheltenham General Hospital, Cheltenham, UK; 6 Department of Health Services Research, University of Liverpool, Liverpool, UK; 7 Public Health England, Cheshire and Merseyside Screening and Immunisation Team, Liverpool, UK

**Keywords:** Diabetic retinopathy, Risk calculation engine, Risk-based screening

## Abstract

**Aims/hypothesis:**

Individualised variable-interval risk-based screening offers better targeting and improved cost-effectiveness in screening for diabetic retinopathy. We developed a generalisable risk calculation engine (RCE) to assign personalised intervals linked to local population characteristics, and explored differences in assignment compared with current practice.

**Methods:**

Data from 5 years of photographic screening and primary care for people with diabetes, screen negative at the first of > 1 episode, were combined in a purpose-built near-real-time warehouse. Covariates were selected from a dataset created using mixed qualitative/quantitative methods. Markov modelling predicted progression to screen-positive (referable diabetic retinopathy) against the local cohort history. Retinopathy grade informed baseline risk and multiple imputation dealt with missing data. Acceptable intervals (6, 12, 24 months) and risk threshold (2.5%) were established with patients and professional end users.

**Results:**

Data were from 11,806 people with diabetes (46,525 episodes, 388 screen-positive). Covariates with sufficient predictive value were: duration of known disease, HbA_1c_, age, systolic BP and total cholesterol. Corrected AUC (95% CIs) were: 6 months 0.88 (0.83, 0.93), 12 months 0.90 (0.87, 0.93) and 24 months 0.91 (0.87, 0.94). Sensitivities/specificities for a 2.5% risk were: 6 months 0.61, 0.93, 12 months 0.67, 0.90 and 24 months 0.82, 0.81. Implementing individualised RCE-based intervals would reduce the proportion of people becoming screen-positive before the allocated screening date by > 50% and the number of episodes by 30%.

**Conclusions/interpretation:**

The Liverpool RCE shows sufficient performance for a local introduction into practice before wider implementation, subject to external validation. This approach offers potential enhancements of screening in improved local applicability, targeting and cost-effectiveness.

**Electronic supplementary material:**

The online version of this article (doi:10.1007/s00125-017-4386-0) contains peer-reviewed but unedited supplementary material, which is available to authorised users.

## Introduction

Systematic screening for sight-threatening diabetic retinopathy (STDR) has been introduced in several European countries and regionally throughout the world, and has been a major driver of improved detection and early treatment. As a doubling of the global prevalence of diabetes mellitus is expected by 2030 [1], with over 10% having STDR [2], there is an urgent need to improve the cost-effectiveness of screening. While current recommendations are for annual screening intervals in most locations [3], there has been a recent move to recommend biennial screening for people with no retinopathy [4–7], including in one systematic review [8], and this was recently endorsed by the UK National Screening Committee [9]. Screening at 3-yearly intervals has been introduced in Sweden, based on data from one programme [10], and is supported as being cost-effective in a recent UK modelling study [11]. However, concerns about the safety and acceptability of extended intervals have held back adoption [12, 13].

Risk engines have been developed in recent years, including in diabetes mellitus for risk of CHD [14], and one has been proposed for diabetic retinopathy [15]. For widespread uptake, reliable flows of data need to be established and designs need to be applicable across a range of populations and health settings.

As part of a programme of research to improve the targeting and cost-effectiveness of screening, we developed a generalisable personalised screening method to allow variable intervals for people with diabetes at high and low risk of developing STDR. We developed and internally validated a risk calculation engine (RCE) to estimate risk of progression to screen-positive or referable diabetic retinopathy and assign individualised screening intervals. We calculated improvement in allocation of screening interval to estimate the effect on number of screen episodes.

## Methods

### Dataset

Data from established digital photographic screening (OptoMize, EMIS Group, Leeds, UK) and primary care systems (EMISweb, EMIS Group) were combined in a purpose-built data warehouse. The local ethics committee approved an opt-out approach to consent (13/NW/0196) and the research was conducted in accordance with the Declaration of Helsinki 2008. Data were collected for all people recorded in primary care as having diabetes mellitus attending the Liverpool Diabetes Eye Screening Programme (LDESP) from the systems used for routine service, anonymised and compiled before transmission to the warehouse.

A set of candidate covariates was selected for the model using patient expert panels and a literature review of known risk factors (see electronic supplementary material [ESM] Methods and ESM Table 1). An RCE development dataset was extracted from the data warehouse containing covariates with ≥ 80% completeness in people with diabetes who were screen negative (non-referable retinopathy) at the first of at least two episodes that occurred in a 5 year sample period. Disease duration was defined as duration of known diabetes mellitus (first recorded date of diabetes or measure of HbA_1c_ in primary care [11]) and assigned at the first screening episode. Values of clinical risk factors prior and nearest to the screen episode date were used.

Screen-positive (the primary outcome) was defined as the presence of any of: multiple blot haemorrhages, venous beading, intra-retinal microvascular abnormalities, new vessels, pre-retinal/vitreous haemorrhage, tractional retinal detachment, exudates within 1 disc diameter (1500 μm) of the foveal centre, group of exudates within the macula or blot haemorrhages within 1 disc diameter of the foveal centre with vision worse than 6/12.

### Model description

**Fig. 1:**
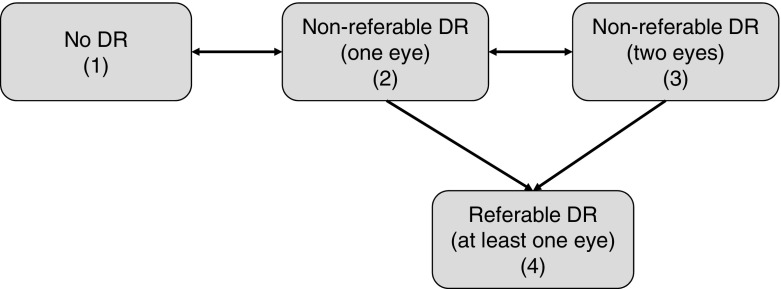
Transitions between diabetic retinopathy disease states of the continuous-time Markov process in the Liverpool RCE. DR, diabetic retinopathy

We selected a continuous-time Markov process to allow for a set of individuals to move independently, or transition, between states over time [16]. The patient state at each time point was defined by level of retinopathy, including separation by one or both eye involvement after Stratton et al [17] (Fig. 1:) (1) no diabetic retinopathy detected; (2) non-referable diabetic retinopathy in one eye only; (3) non-referable diabetic retinopathy in both eyes; and (4) referable diabetic retinopathy (screen-positive for at least one eye). Only one baseline screening event was used.

The risks, or intensities, for each transition were entered into the model within a probability matrix containing Weibull transition intensities estimated from the data [18–20]. A detailed description is provided in the ESM Methods.

The data in the RCE development dataset is an example of panel data [16, 18] where information on an individual’s disease is sampled at time points not typically coincident with the change in disease state. This interval-censoring problem is illustrated in ESM Fig. 1 and required special methods. Missing clinical data were handled using multiple imputation [21] (ESM Methods) repeated ten times to properly account for variability due to unknown values.

### Model fitting and covariate selection

Covariates meeting the above criteria were ranked using Wald statistics. A set of nested models were built to estimate corrected Akaike’s information criterion (AICc). This method combines estimation (i.e. maximum likelihood) and model selection under a unified framework [16, 22, 23]. AIC was corrected to adjust for the number of covariates (see ESM Methods); this is a method which aims to reduce the effect of overfitting by applying a penalty for model complexity. The model with the smallest AICc was chosen to give the best fit to the data.

### Patient expert group

A patient involvement group was developed through local and national patient groups and local advertisements with a mix of backgrounds, sex and diabetes type. The group developed their knowledge of disease and patient pathways and the assessment of risk over several tailored sessions. At the end of these, they expressed that they had sufficient knowledge to give considered input into the study design. Acceptability and feasibility were considered for the application of the RCE output for a range of risk thresholds and alternative screen intervals.

### Data validation and model checking

We checked the development dataset using random samples of event vectors which were independently checked manually and programmatically. The model was checked for influence of outliers, regression and distributional assumptions, and Pearson-type goodness-of-fit and corrected C-index were calculated.

Bootstrapping (to estimate the optimism of validation measures) and fourfold cross-validation were used for internal validation (see ESM Methods). Further internal validation was conducted using a geographical split based on the deprivation index [24], to assess whether the performances of the model were unduly affected by extremes of prevalence rates of positive screening events. Areas under the receiver operating curves were calculated as overall indicators of sensitivities and specificities.

### Implementation

The effect of a set of risk thresholds (5%, 2.5%, 1%) on screening-interval allocation was investigated using the fourfold validation sets described above, and a final threshold selected in discussion with the patient expert group. The proportion of screen-positive events that occurred before the allocated interval was calculated for each risk threshold. Overall numbers of screening episodes were calculated over a 2 year period and compared with an annual programme.

Predictions in a clinical environment (using the fitted model) for new observations with missing covariates were obtained by a simple imputation strategy: we replaced the missing values of each covariate with a 75th percentile value estimated from full data at first screening to give a ‘worst case’ prediction.

A small sample of cases assigned by the RCE to 6, 12 and 24 months rescreen interval were independently checked against patient records for clinical credibility.

## Results

The data repository contained 2.48 × 10^10^ data fields across 302 covariates. Data extracted into the RCE development dataset were from 11,806 people with diabetes actively attending the LDESP between 20 Feb 2009 and 4 Feb 2014 (46,525 episodes, 388 screen-positive events). Data flow is shown in ESM Fig. 2.

Covariates that met the entry criteria are listed in Table 1. Those that gave the best fit (total rescaled AICc = 0) and were included in the final model were: disease duration, HbA_1c_, age at diagnosis, systolic BP and total cholesterol. Although the retinopathy stage is not technically a covariate, it is included in the table to show the improvement in predictive power when covariates are added to the model.

**Table 1:** Ten covariates included in the initial model with corresponding Wald statistics, rescaled corrected AICc and proportions of explained likelihood

Covariate	Wald statistic	Rescaled AIC_c_	% Explained likelihood
Retinopathy (baseline)^a^		893.65	–
+ Disease duration (years)^a^	293.4	423.23	48
+ HbA_1c_ (mmol/mol)^a^	201.2	68.61	85
+ Age at diagnosis (years)^a^	44.2	10.85	92
+ Systolic BP (mmHg)^a^	18.9	6.61	94
+ Total cholesterol (mmol/l)^a^	18.7	0	96
+ Disease type	15.2	0.99	97.5
+ Diastolic BP (mmHg)	8.2	5.61	98.6
+ eGFR (ml min^−1^ 1.73 m^−2^)	5.4	13.63	99.4
+ Sex	5.1	24.95	99.9
+ HDL-cholesterol (mmol/l)	0.73	40.99	–

^a^Covariates included in the final model

The risk model is summarised in the following three equations. The first expression gives the hazard rates (or intensities, or ‘risks’) of going from one state to another, for each of the six transitions shown in Fig. 1, each one dependent on the full set of covariates and the full local population history:

1 $$ \log {\lambda}_{ij}={\beta}_{ij}^0+{\beta}_{ij}^{AgeD} AgeD+{\beta}_{ij}^{DiseaseD} DiseaseD+\cdots +{\beta}_{ij}^{SP} SBP $$

where *i* , *j* = {1, 2, 3, 4} and ${\beta}_{ij}^C$ is the model parameter for covariate *C* (or baseline intensity when *C* = 0); AgeD is age at diagnosis, DiseaseD is disease duration.

From the hazard rates function (1), a transition intensity matrix is derived for the four states (each row sums to 0):

2 $$ Q=\left({\displaystyle \begin{array}{cccc}\hfill -{\lambda}_{11}\hfill & \hfill {\lambda}_{12}\hfill & \hfill 0\hfill & \hfill 0\hfill \\ {}\hfill {\lambda}_{21}\hfill & \hfill -{\lambda}_{21}-{\lambda}_{23}-{\lambda}_{24}\hfill & \hfill {\lambda}_{23}\hfill & \hfill {\lambda}_{24}\hfill \\ {}\hfill 0\hfill & \hfill {\lambda}_{32}\hfill & \hfill -{\lambda}_{32}-{\lambda}_{34}\hfill & \hfill {\lambda}_{34}\hfill \\ {}\hfill 0\hfill & \hfill 0\hfill & \hfill 0\hfill & \hfill 0\hfill \end{array}}\right) $$

ESM Fig. 3 shows the two baseline transition intensities to the screen-positive state in the rightmost column of Eq. (2). Probabilities of transition occurring at a specific time *t* are obtained by using the following equation:

3 $$ P(t)=\exp \left({Qt}^{0.9}\right) $$

where the exp() operator is the matrix exponential and *α* = 0.9 is the estimated Weibull shape parameter. The shape parameter 0.9 reflects the clinical observation that a person is more likely to move between disease states earlier rather than later.

Table 2 shows the estimated baseline hazard ratios, with 95% CIs. In Table 3, we report the estimated baseline probabilities of each state transition, with 95% CIs.

**Table 2:** Baseline hazard ratios for each transition

Transition	AgeD	DiseaseD	HbA_1c_	Chol	SBP
1 → 2	1.00450 (1.00115, 1.00787)	1.0280 (1.0213, 1.0348)	1.0101 (1.00743, 1.0128)	0.963 (0.923, 1.00521)	1.00409 (1.00104, 0.0073)
2 → 1	1.00580 (1.00237, 1.00919)	0.983 (0.975, 0.992)	0.998 (0.995, 1.00140)	1.0153 (0.973, 1.0592)	0.999 (0.996, 1.00244)
2 → 3	0.989 (0.984, 0.994)	1.0261 (1.0173, 1.0350)	1.00621 (1.00221, 1.0102)	0.965 (0.901, 1.0333)	0.998 (0.993, 1.00255)
2 → 4	1.0245 (0.990, 1.0605)	0.989 (0.931, 1.0510)	1.00554 (0.983, 1.0285)	1.0231 (−0.27, 0.37)	1.00342 (0.977, 1.0310)
3 → 2	1.00839 (1.00329, 1.0135)	0.959 (0.949, 0.968)	0.990 (0.985, 0.994)	1.0836 (1.0147, 1.157)	0.997 (0.993, 1.00126)
3 → 4	0.986 (0.977, 0.995)	1.00420 (0.989, 1.0200)	1.0164 (1.00888, 1.0239)	1.0346 (0.918, 1.166)	1.00501 (0.996, 1.0141)

95% CIs shown

AgeD, age at diagnosis

Chol, cholesterol

DiseaseD, disease duration

SBP, systolic BP

**Table 3:** Baseline probabilities of state transition at 1 year

Transition	Probability
1 → 2	0.114 (0.111, 0.118)
2 → 1	0.552 (0.541, 0.565)
2 → 3	0.141 (0.134, 0.148)
2 → 4	0.0163 (0.0139, 0.0202)
3 → 2	0.283 (0.272, 0.294)
3 → 4	0.0574 (0.0485, 0.0678)

95% CIs shown

Further details are given in ESM Methods.

### Data and model checking

The pseudo-likelihood ratio *p* value for the summary residuals vs time was 0.04, suggesting linearity to hold between 0 and 2 years, with a possible lack of fit beyond 2 years (ESM Fig. 4). Although the *p* value was below 0.05, there is not enough evidence of a lack of fit because of the small number of events relative to the model complexity [20, 22]. Cox–Snell residuals are shown in ESM Fig. 5: the calibration curve was close to the theoretical optimal calibration, showing that the model tended to give slightly pessimistic predictions of failure. The Pearson-type statistic for the Liverpool RCE model was 0.57, denoting not enough evidence to reject the null hypothesis of good fit. Cross-validation showed only very small effects, i.e. the training and test performance measures were essentially the same. Fitting the model to the most deprived 65% of individuals produced only very small changes in risk allocation of the non-deprived group.

The patient expert panel identified acceptable screen intervals of 6, 12 and 24 months and risks of 1% and 2.5% as acceptable risks of missing screen-positive disease at any future screen episode. Exploration of the effect of different risk thresholds on allocation to the three different screen intervals using our four-way cross-validation is shown in Table 4. As the risk threshold decreased, the proportion of incorrect screen-interval allocations decreased for screen positives (overestimation) and increased (underestimation) for screen negatives.

**Table 4:** Analysis of the effect of allocation of screening interval by the RCE compared with annual interval

	Screening			
Variable	Individualised based on the Liverpool RCE			Annual
	Risk threshold			
	5%	2.5%	1%	
Screening interval^a^				
6 months	5.5%	10.7%	26.3%	0%
12 months	4.4%	8.6%	9.3%	100%
24 months	90.1%	80.7%	64.4%	0%
Screen-positive (%)^b^				
Correct allocation: events occurring after the predicted screening date	65.6%	78.0%	91.1%	49.8%
Incorrect allocation: events occurring before the predicted screening date (overestimated)	34.4%	22.0%	8.9%	50.2%
Screen negative (%)^b^				
Correct allocation^c^	90.4%	79.8%	62.2%	0%
Incorrect allocation: screening date given ‘too early’ (underestimated)	9.6%	20.2%	37.8%	100%
Proportion of reduction in visits compared with annual interval	39.5%	29.7%	5.9%	Reference value

^a^Proportion of individuals allocated to 6, 12 and 24 month screening using 5%, 2.5% and 1% risk thresholds

^b^Distribution of screen-positive and -negative events depending on risk threshold is also shown, followed by the proportion of reduction of visits compared with annual screening

^c^Excluded data from individuals with insufficient follow-up data

For an annual interval, the overestimation was 50.2% and underestimation 100%; when compared with this interval, the number of individuals correctly allocated was greater for the model with any of the three thresholds. The number of intervals which were either over- or underestimated was also lower for the model at all three thresholds. Table 5 shows the detailed comparison between the overall numbers of screening episodes in an annual programme and the Liverpool RCE model. For all three risk thresholds, there was a reduction in the overall number of screening episodes required (summarised in Table 4). The research team and patient expert panel considered that a 2.5% criterion showed a satisfactory distribution across the three screening intervals and a reasonable reduction in episodes, and this was selected for implementation.

**Table 5:** Screening episodes required over a 2 year period from each validation set (and combined) according to threshold, and comparison with annual screening

Specification/model allocation	Number of screening episodes required in a 2 year period					Difference from standard allocation (%)
	6 months^a^	12 months^a^	24 months^a^	Total	Standard allocation^b^	
5%						
VS1	728	238	2699	3665	6000	−2335 (−38.9)
VS2	668	262	2702	3632	6000	−2368 (−39.5)
VS3	712	274	2685	3671	6000	−2329 (−38.8)
VS4	548	288	2719	3555	6000	−2445 (−40.8)
Overall	2656	1062	10,805	14,523	24,000	−9477 (−39.5)
2.5%						
VS1	1276	460	2451	4187	6000	−1813 (−30.2)
VS2	1280	526	2417	4223	6000	−1777 (−29.6)
VS3	1308	544	2401	4253	6000	−1747 (−29.1)
VS4	1260	540	2415	4215	6000	−1785 (−29.8)
Overall	5124	2070	9684	16,878	24,000	−7122 (−29.7)
1%						
VS1	3140	546	1942	5628	6000	−372 (−6.2)
VS2	3124	584	1927	5635	6000	−365 (−6.1)
VS3	3208	548	1924	5680	6000	−320 (−5.3)
VS4	3136	560	1936	5632	6000	−368 (−6.1)
Overall	12,608	2238	7729	22,575	24,000	−1425 (−5.9)

Breakdown of numbers of screening episodes required over a 2 year period from each validation set (and combined) if the cut-off was 5%, 2.5% or 1% and the midpoint of positive-screen event interval was truly representative of when that positive event actually occurred, along with a comparison with the currently used standard annual screening allocation

^a^Number of screening episodes at 6, 12 and 24 months is calculated as 4×, 2× and 1× the number of people allocated to those screening episodes, respectively, to represent the number of screenings they would attend over a 2 year period

^b^Standard number of screening episodes based on all individuals given annual screening (i.e. 2× number of individuals)

VS, validation set

Using the 2.5% threshold, the corrected C-index for the model was 0.687 and corrected AUCs (with 95% CIs) were 0.88 (0.83, 0.93) at 6 months, 0.90 (0.87, 0.93) at 12 months and 0.91 (0.87, 0.94) at 24 months. The four-way random data split gave sensitivities and specificities for a risk threshold of 2.5% at 6, 12 and 24 months, respectively: 6 months 0.61, 0.93; 12 months 0.67, 0.90; and 24 months 0.82, 0.81.

Clinical review of sampled cases (*n* = 18) indicated that allocations to individualised screen intervals appeared reasonable.

## Discussion

We have developed and tested an RCE in which an individual’s risk can be predicted from contemporaneous routinely collected clinical data, referenced to the clinical histories of the local population, using covariates of local relevance. The risk can be reassessed at each screening episode as new clinical information is acquired.

The Markov approach we have used allows a dynamic model of the retinopathy history to be built. In a sense, the model ‘compresses’ the information about time evolution. The Markov property can be summarised by the phrase ‘The future is predicted from the past through the present’, and is particularly appropriate to our setting.

The strengths of our model include our approach to tackling the data in routine screening. Retinopathy data in screening is interval censored [16] in that the event seems as if it has happened when it is detected. This may lead to biased estimates, as it ‘seems’ like the disease developed later than it actually did. Unlike other ‘classic’ model types, including the Cox model, the Markov approach can internally handle this interval censoring. In addition, it predicts the probabilities of transition for all disease states. ‘Real life’ data from routine clinical practice inevitably introduces missingness and recording errors. We embedded a model for multiple imputation of missing covariates, which was required to allow our RCE to run effectively.

Potential limitations of our RCE relate to model design and some of the covariates. We did not adjust for misclassification of retinopathy during grading. This could be addressed by adding a misclassification model, but at the cost of substantially more observations and computational complexity. Some covariates were not informative in the Liverpool setting. Ethnic diversity is low and the prevalence of abnormal eGFRs <60 ml min^−1^ 1.73 m^−2^ was only 14.5%. Other covariates such as social deprivation score may be worth adding. ‘Type of diabetes’ may not be accurately recorded in primary care and the increased use of insulin in type 2 diabetes makes ‘insulin usage’ an unreliable criterion. We used date of first HbA_1c_ test to improve data on ‘duration of diabetes’, helpful especially in people with long durations, but less reliable since the introduction of HbA_1c_ as a primary screening test.

The model consistently showed good levels of prediction for the 2.5% risk threshold. The numbers of screen-positive cases with overestimated screening dates and screen-negative cases with underestimated screening dates were reduced. The majority of people were correctly allocated (78% of screen positives, 80% of screen negatives), with a reasonable allocation of (approximately) 10%:10%:80% across the 6, 12 and 24 month intervals. The number of patients who had the screen event before the allocated screening date was reduced by more than half and the overall number of screening episodes was reduced by 30%.

We included a strongly embedded local patient group, which allowed us to develop an appropriate preliminary covariate list and acceptable screen intervals and risk threshold. This group developed expertise over a series of meetings and provided substantial input into design and implementation. Strong patient and professional involvement is very valuable in study design and delivery.

Our RCE development process is suitable for a wide range of geographical locations and populations with a minimum prerequisite of a centrally maintained disease register with adequate historical data. Revision/addition of covariates can be accommodated based on the strength they add to a locally developed model. For example, higher prevalence of poor diabetes control or renal disease may strengthen the effect of HbA_1c_ or eGFR. Alternative intervals including extension beyond 24 months could be developed subject to acceptability. Local populations may select alternative risk thresholds depending on the perception of risk. We give the key steps to developing and building such a system in the text box.
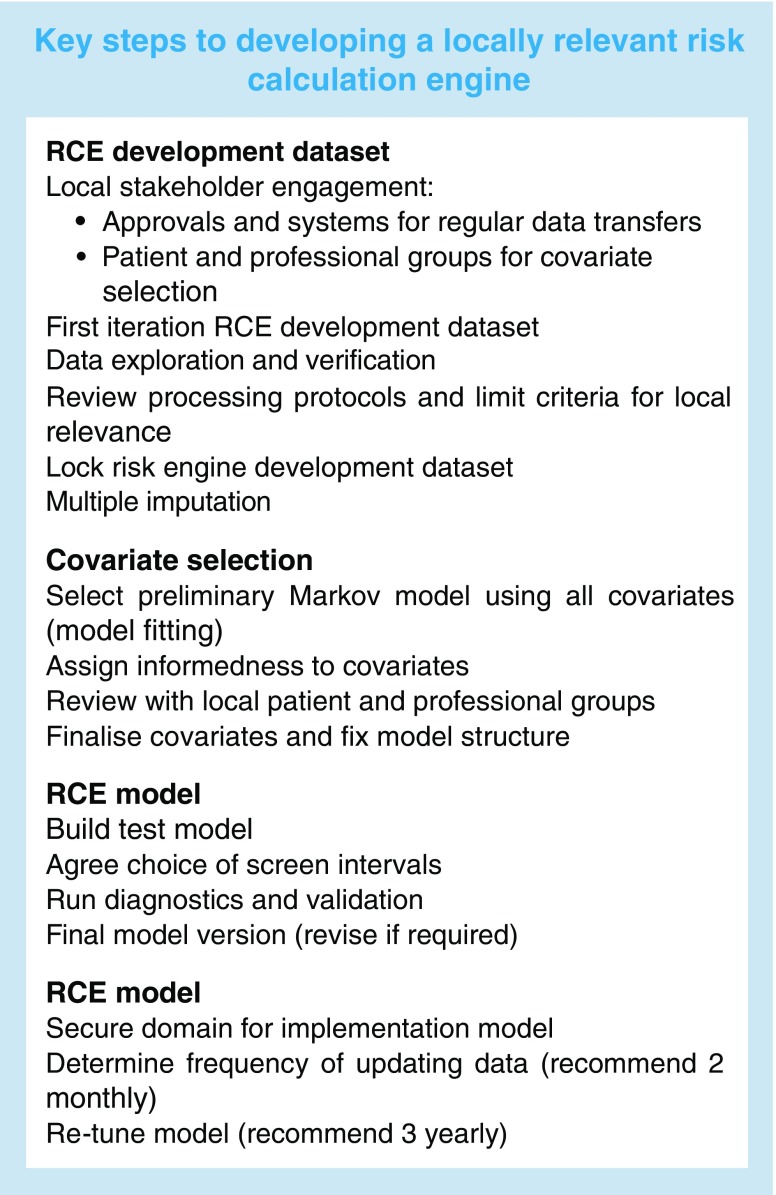


The use of near-real-time data and a model developed from local data in our approach is novel. Aspelund et al developed a risk-estimating model in Iceland [15]^.^ They used a proportional hazards Weibull model informed by local retinopathy data between 1994 and 1997 and risks for covariates estimated from data published in the 1990s. ROC analysis showed a fair performance, with 59% fewer visits than annual screening. Van der Heijden et al tested this model in an up-to-date prospective cohort of people with type 2 diabetes [25]. Of a total of 8303, 3319 met the eligibility criteria, with a mean of 53 months follow-up. Discriminatory ability was good (C-statistic 0.83), but 67 of 76 people (88.2%) who developed STDR developed it after the time predicted by the model. This overestimation of risk highlights the weakness of using historical data.

Hippisley-Cox and Coupland recently developed equations to predict 10 year rates of amputation and blindness using similar methods to us [26]. They studied routinely collected general practice and hospital episode data from 454,575 people with diabetes. A web-based 10 year calculator using Cox’s proportional hazards models was developed. They reported comparable C-statistics (≥ 0.73) and conducted external validation using 357 practices that used a different database. The principal limitation of this large study was the lack of validation of the diagnosis of blindness.

Risk engines have been developed in other diseases including coronary heart disease, stroke and lipid therapy. The UK Prospective Diabetes Study developed a risk engine for predicting coronary heart disease [14], now in its second version (UKPDS Outcomes Model 2).

We included clinical risk factors in our model. It has recently been suggested that retinopathy data are sufficient to develop a risk stratification to extend screening intervals for people at low risk [27]. This may prove to be a reasonable and pragmatic approach. We had to overcome significant challenges in developing a near-real-time data flow; this may be too difficult in some populations. However, we determined that including clinical data would aid acceptance amongst the professional community, offer better prospects for generalisability and allow inclusion of more frequent screening for high-risk individuals. Our view is supported by our own data [28] and those of others [29], and also by our patient expert group. We do recognise that, as yet, estimates of resource requirements for the effective introduction of our type of RCE are not available.

External validation of models is required before general implementation [30]. However, validation methods for an approach such as ours are not well developed. An RCE comprises two principal components: (1) the dataset containing a set of covariates and the outcome of interest; and (2) the mathematical model applied to the data in the dataset. The application to a population is specific to that population. In addition to the interval censoring described above, screening data are also not proportional. This makes problematic the use of widely accepted statistics for assessing effectiveness of diagnostic tools based on Kaplan–Meier methods. An approach to validation was developed, taking these constraints into account, comprising dataset validation, model checking, internal validation (including data splitting, bootstrapping, C-index) and estimation of sensitivities/specificities at specified intervals, all recognised internal validation methods [30]. An implementation phase will include model updating (temporal validation and model tuning) and the opportunity for comparative cross population (external) validation to correct for potential overperformance [31].

We believe that the Liverpool RCE is feasible, reliable, safe and acceptable to patients. Implementation of our RCE into routine clinical practice would offer potentially significant transfer of resources into targeting high-risk and hard-to-reach groups and improved cost-effectiveness. Based on the internal validations we have performed, it shows sufficient performance for a local introduction. However, wider implementation will require an external validation process and testing of safety and acceptability in an RCT setting [31]. Investment in IT systems will be required for implementation in large-scale health systems, such as the NHS, and to support further validation.

## Electronic supplementary material

ESM (PDF 556 kb)

## Abbreviations

AICc: Corrected Akaike’s information criterion
LDESP: Liverpool Diabetes Eye Screening Programme
RCE: Risk calculation engine
STDR: Sight-threatening diabetic retinopathy
