# Supplementary material for: Individualised variable-interval risk-based screening for sight-threatening diabetic retinopathy: the Liverpool Risk Calculation Engine
Source: Diabetologia. 2017 Aug 24;60(11):2174–82. doi: 10.1007/s00125-017-4386-0 (PMC6448900; doi:10.1007/s00125-017-4386-0)
Supplement: Supplementary file 1 — (PDF 556 kb) [file 125_2017_4386_MOESM1_ESM.pdf]

Eleuteri et al

Electronic Supplementary Material

ESM Methods

Candidate covariates
302 variables were identified in the data warehouse derived from the photographic screening (OptoMize, Digital Healthcare Ltd.), primary care (EMISweb, EMIS Healthcare Ltd.) and secondary care integrated patient management systems in the following domains: demography, national diabetic retinopathy grading classification, feature specific grading, visual acuity, clinical risk factors, drug treatment, hospital attendance, hospital treatment episodes. Covariates were reviewed by a patient expert panel and review of the literature and extracted in a RCE Development Dataset (listed in ESM Table 1).

Some covariates were unsuitable for inclusion. In the Markov model structure that we selected the model is built upon the histories of the patients. Attendance was therefore implicit in the history of each patient and could not function as an independent covariate. Social deprivation was based on GP postcode and was therefore unsuitable at in individual level.

Continuous-time Markov process [16]
The process considers a set of individuals independently moving among k states (example in Figure 1), denoted by $1,\ldots,k$. If $t_{n-1} < t_n$ then, if we denote by $X(t)$ the state occupied at time t by an individual is:

$$\Pr\{X(t_n) £ x_n \mid X(t), t £ t_{n-1}\} = \Pr\{X(t_n) £ x_n \mid X(t_{n-1})\} \quad (1)$$

and more generally, if $t_1 < t_2 < L \ < t_n$ then:

$$\Pr\{X(t_n) \le x_n \mid X(t_{n-1}),\ldots, X(t_1)\} = \Pr\{X(t_n) \le x_n \mid X(t_{n-1})\} \quad (2)$$

Let P be the transition probability matrix with entries:

$$p_{ij}(s,t) = \Pr\{X(t) = j \mid X(s) = i\} \quad (3)$$

for i, j=1,…,k. The model is specified in terms of transition intensities (or hazards or risks) [18,19]:

$$\lambda_{ij}(t) = \lim_{\Delta t \to 0} p_{ij}(t, t+\Delta t)/\Delta t, \quad i \neq j \quad (4)$$

We also defined:

$$\lambda_{ii}(t) = -\sum_{i \neq j} \lambda_{ij}(t), \quad i = 1,\ldots,k$$

so we could define a $k \times k$ transition intensity matrix $Q(t)$ with entries $l_{ij}(t)$ (see Equation 2 in main manuscript). The simplest model assumes that $\lambda_{ij}(t) = \lambda_{ij}$ is independent of t, which implies transition intensities from an exponential distribution. In this case the process is stationary [16], that is given two time points, s and t, the process will only depend on the difference t-s.

We can consider a simple nonhomogeneous Markov model [17] with a time-dependent intensity matrix of the form $Q(t) = Q_0 g(t,\alpha)$ where Q_0 is a fixed transition intensity matrix. In this case, $g(t,\alpha)$ defines an operational time such that the process is time-homogeneous Markov. For a given α, let $s = \int_0^t g(u,\alpha)du$ and define $Y(s) = X(t)$, then $Y(s)$ is a homogeneous Markov process with intensity matrix Q_0.

In our model we consider Weibull transition intensities with shape parameter α, given by $g(t,\alpha) = \alpha t^{\alpha-1}$. The operational time in this case is $s = t^{\alpha}$, which is a

simple power transformation of the observed times (note that we recover the time-homogeneous model when $\alpha = 1$). Following Kalbfleisch and Lawless [18], we estimate α from the data, by maximising the maximised model log-likelihood with respect to it by a simple line-search. Introduction of the α parameter in the model increases the overall likelihood, even considering the increased model complexity, so it improves the overall quality of the fit.

A relationship between transition intensities and transition probabilities is given by the following relationship and expressed as a matrix [18,19]:

$$P(t) = \exp(Qt) \quad (5)$$

which is the solution of the Kolmogorov equation $\mathrm{d}P(t) = P(t)Q\,\mathrm{d}t$.

The equality provides estimates of the state transition probabilities. Note that the exponential operation is to be intended as a matrix operation, which is in general computationally complex. We assumed the entries of the matrix Q to be dependent on b functionally independent parameters, which might represent the baseline transitions of the model, and/or regression parameters relating the instantaneous transition intensities to a set of covariates. If each individual under study has associated a covariates vector of r risk factors $\mathbf{z}$, we assume the following form for the transition intensities of the Markov process:

$$\log \lambda_{ij}(\mathbf{z}) = \beta_{ij}^{0} + \beta_{ij}^{1} z_1 + \beta_{ij}^{2} z_2 + \ldots + \beta_{ij}^{r} z_r \quad (6)$$

where β_{ij}^{0} is the log of the baseline transition intensity (we assume the baseline corresponds to the mean of the covariates). The covariates vector can be time-dependent, representing the history of the risk factors in a time interval, or a suitable summary statistic of the history, or fixed at baseline.

If we observe a random sample of n individuals at times $t_0, t_1, \ldots, t_m$, and denote by n_{ijl} the number of individuals in state i at t_{l-1} and j at t_l, then it can be shown that conditional on the distribution of individuals among states at t_0, the likelihood function for the parameters $\beta_1, \ldots, \beta_b$ can be written as:

$$L(\beta_1, \ldots, \beta_b) = \prod_{l=1}^{m} \prod_{i,j=1}^{k} p_{ij}(t_l - t_{l-1}; \beta_1, \ldots, \beta_b)^{n_{ijl}} \quad (7)$$

Note that the transition probabilities in the likelihood depend (typically nonlinearly) on the parameters through the Kolmogorov equation [18]. Maximisation of the above likelihood function with respect to the parameters provides estimates of the entries of the transition intensity matrix.

It should be noted that, in general, information on an individual's passage through the disease states usually would not be complete, in the sense that we will only know an individual's status at several points in time as illustrated in Figure 1 [19].

The model was fitted using the *msm* library of the R package, with additional code written by AE to interface the library with the multiple imputation functions and the covariate selection procedures (see next sections).

Imputation in the model fitting

Imputing missing values and then doing an ordinary analysis as if the imputed values were real measurements is usually better than excluding subjects with incomplete data. Problems with case-wise deletion of subjects with missing values include a reduction of the sample size, an increase in the real standard error of parameter estimates, and biased parameter estimates if data is not missing completely at random. Multiple imputation doesn't incur any of the above problems; however, methods for properly accounting for having incomplete data can be complex.

Multiple imputation uses random draws from the conditional distribution of the target variable, given the other variables (e.g., we draw a value of HbA_{1c}, conditional on

cholesterol, age at diagnosis, sex etc.). Note that causal chains are not relevant, so we can use variables measured “in the future” (both for the same or a different subject’s history). Conditioning on outcomes is also possible in multiple imputation procedures, and it helps reduce the bias in parameter estimates; in our model, we conditioned on both screening times and states.

To properly account for variability due to unknown values, the imputation is repeated M times, where $M \geq 3$ (for our model we have M =10.) Each repetition results in a “completed” dataset that is analysed using the standard method. Parameter estimates are averaged over these multiple imputations to obtain better estimates than those from single imputation. The variance-covariance matrix of the averaged parameter estimates, adjusted for variability due to imputation, is estimated using the following [21,22]:

$$V = \frac{1}{M}\sum_{i=1}^{M} V_i + \frac{M+1}{M} B \quad (8)$$

where V_i is the ordinary complete data estimate of the variance-covariance matrix for the model parameters from the ith imputation, and B is the between-imputation sample covariance matrix, the diagonal entries of which are the ordinary sample variances of the M parameter estimates.

We used the aregImpute multiple imputation algorithm, implemented in the *aregImpute* function, part of the *rms* library of the R package. In the following we give a necessarily brief description of the algorithm; for further details, see the documentation of the function, and the previously referenced sources.
aregImpute takes all aspects of uncertainty into account using the bootstrap to approximate the drawing of predicted values and using different bootstrap samples for each multiple imputation. aregImpute applies weighted predictive mean matching so that no distributional assumptions are required. We used van Buuren’s “Type 1” matching [21] to capture the right amount of uncertainty: here one computes predicted values for missing values using a regression fit on the bootstrap sample, and finds donor observations by matching those predictions to predictions from potential donors using the regression fit from the original sample of complete observations.

When a predictor of the target variable is missing, it is first imputed from its last imputation when it was a target variable. A donor is defined as a complete observation whose predicted target is closest to the predicted value of the target from all complete observations.

Covariate selection
Estimation and model selection were combined under a unified framework achieved by minimisation of:

$$\mathrm{AIC} = -2\log \mathrm{Likelihood}(\hat{\theta} \mid \mathrm{data}) + 2K \quad (9)$$

where K is a measure of the complexity of the model and for a linear model, is the number of parameters that enter the model. Because n/K<40 (where n=388 is the number of screen positive events, and K=37, including the α parameter to model nonhomogeneity) the expression was revised (after Harrell [22]) giving the “corrected” second-order AIC [16,20,31]:

$$\mathrm{AIC}_C = -2\log \mathrm{Likelihood}(\hat{\theta} \mid \mathrm{data}) + 2K\left(1 + \frac{K+1}{n-K-1}\right) \quad (10)$$

Because individual AIC values are not interpretable due to containing arbitrary constants and being much affected by sample size, AIC_c was rescaled to:

$$\Delta_i = \mathrm{AIC}_i - \mathrm{AIC}_{\min} \quad (11)$$

where $\mathrm{AIC}_{\min}$ is the minimum of the different AIC_c values. Δ_i was then interpreted as the information loss experienced if we used fitted model g_i rather than the best fitting model. The covariates retained in the model were the ones that achieved a total rescaled AIC_c=0.

Model checking
Note that due to the multiple imputation process, both bootstrapping and 4-fold cross validation were applied to the “median” dataset (i.e. the data set obtained by replacing all missing covariates with the medians over the ten imputation sets).

ESM Results

Covariate selection and risk model
Covariates were centered at their means, so the baseline transitions refer to a hypothetical subject with mean covariate values: age at diagnosis: 61 years; time since diagnosis of diabetic disease: 6 years; HbA_{1c}: 7.2% (55.5 mmol/mol); total cholesterol: 4.3 mmol/l; systolic blood pressure: 132 mmHg.

As described above missing entries were imputed using multiple imputation techniques. All covariates (including those that didn’t enter the final model) and the outcomes (screening times and associated states) were used in the imputation process. Missing entries for the covariates used in the model fitting (see Table 1) for which multiple imputation was required were: disease duration 994; HbA_{1c} 6311; systolic BP 4651; total cholesterol, 7615; diastolic BP 4643; eGFR 8271; HDL cholesterol 8303

The two baseline transition intensities to the screen positive state in the rightmost column of Q equation (2)) are shown in ESM Figure 3.

ESM Table 1 Candidate covariates developed by the patient expert panel and literature review.

Data type	Name
Retinopathy	RxMx[a] baseline
Demographic	Age (years)
	Gender
	Ethnicity
Systemic risk factors	Type of diabetes
	Known duration of diabetes (years)
	HbA_{1c} (mmol/mol)
	Smoking
	BMI (kg/m^2)
	Total cholesterol (mmol/l)
	HDL cholesterol (mmol/l)
	LDL cholesterol (mmol/l)
	Systolic blood pressure (mmHg)
	Diastolic blood pressure (mmHg)
	eGFR (ml min^{-1} $1.73m^{2\ -1}$)
	Albumen creatinine ratio (mmol/l)
	Triglycerides (mmol/l)
	Insulin use
	Medications

[a] National Diabetic Eye Screening Programme grading classification

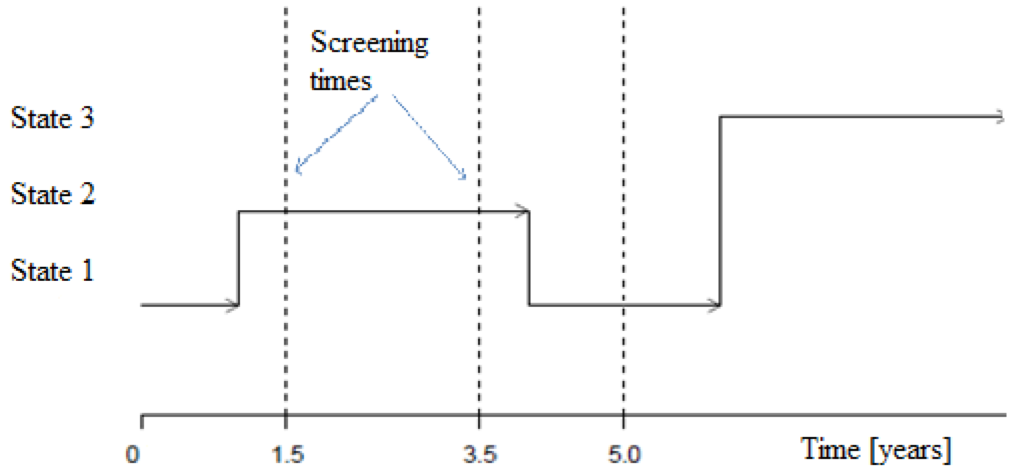

ESM Fig. 1 Example of panel or interval censored data as applied to screening data in ISDR dataset. The process in this example is observed on three occasions, at times 1.5, 3.5 and 5 years, in the states 2, 2 and 1, respectively. No transition is observed exactly (and state 3 is not observed at all).

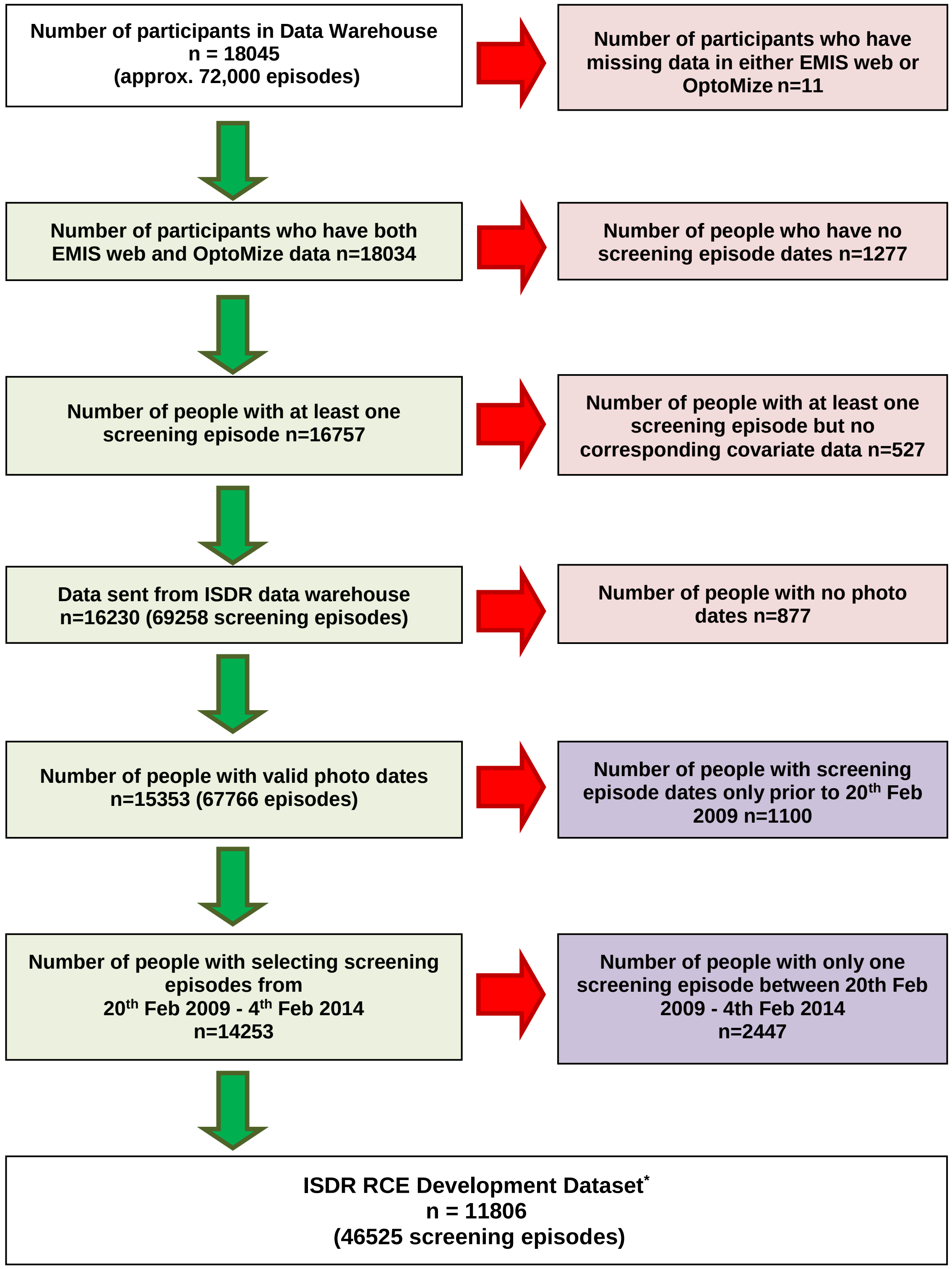

* ISDR RCE Development Dataset contains: 11806 participants (46525 screening episodes) who have at least 2 screening episodes from 20th Feb 2009 - 4th Feb 2014 and whose 1st screening episode was negative in both eyes (i.e. not R2, R3 or M1)

ESM Fig 2

Consort style diagram showing the distribution of subjects forming the ISDR RCE Development Dataset

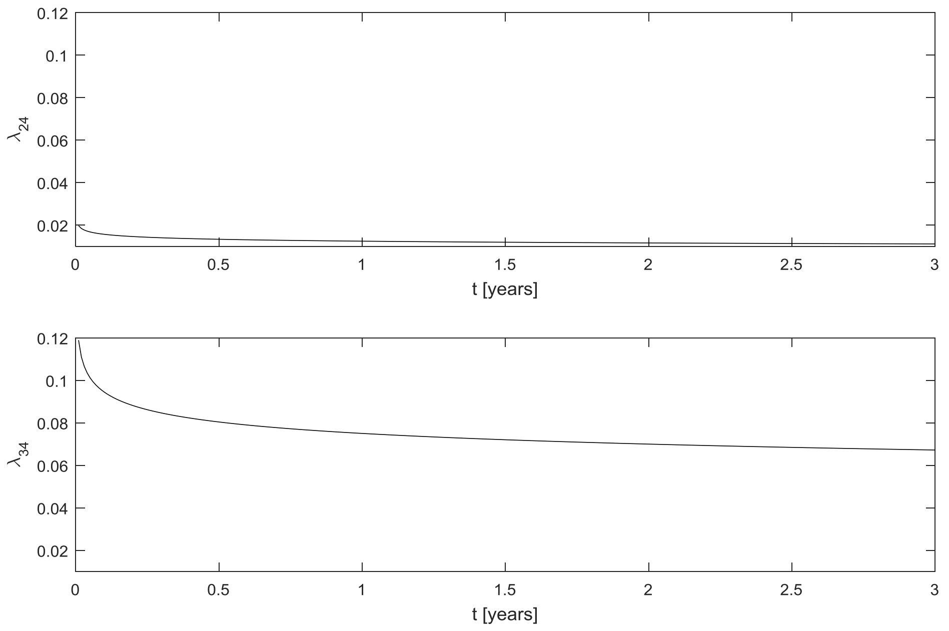

ESM Fig. 3: Transition intensity baselines to the screen positive state from the non-deferable retinopathy, one eye (top) and from the non-deferable retinopathy, two eyes (bottom) states.

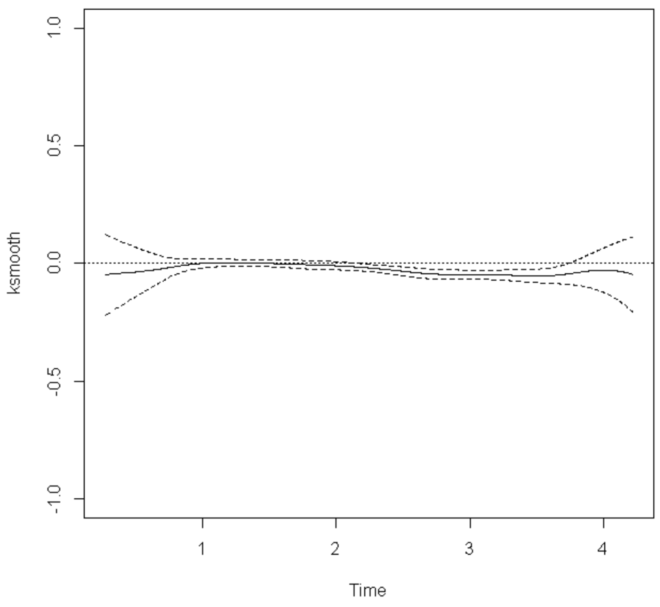

ESM Fig. 4: Smoothed summary residual (y axis) vs. follow up time (x axis, units in years) with 95% confidence interval used to check time homogeneity in the Liverpool Risk Calculation Engine.

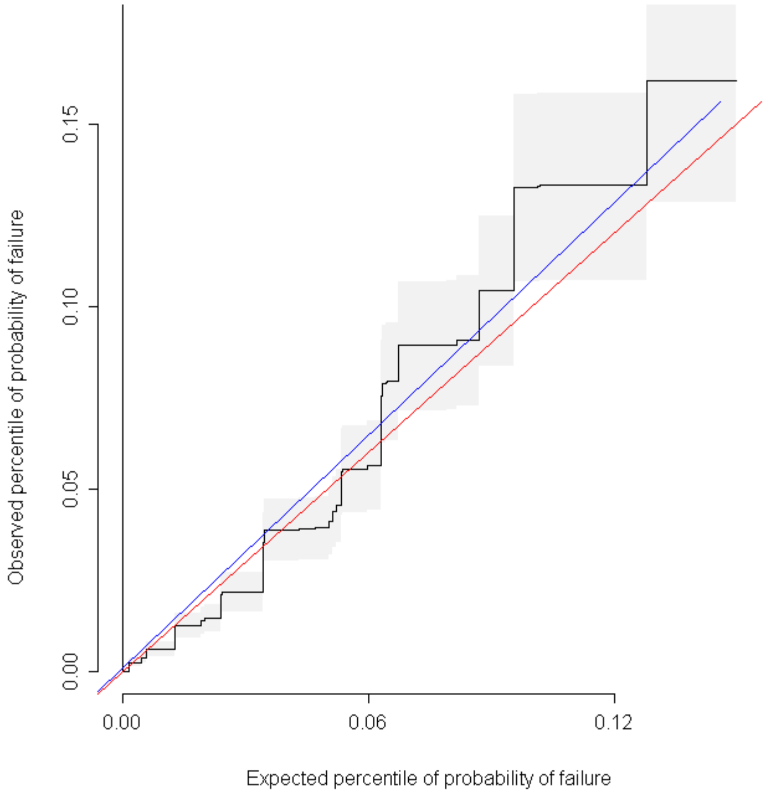

ESM Fig. 5. Cox-Snell residuals for the Liverpool RCE model. The black line shows the Turnbull estimate of the calibration curve; the blue line is a smoothing of the black line, and the red line represents the theoretical calibration. The calibration curve is close to the theoretical optimal calibration, and it shows the model tends to give slightly pessimistic predictions of failure.

ISDR Study Group

Simon P Harding (Study Group Chair), Deborah M Broadbent, Anthony C Fisher, Mark Gabbay, Marta García-Fiñana, Marilyn James, Tracy Moitt, John R Roberts, Daniel Seddon, Irene M Stratton, Paula Williamson; Lola Awoyale, Ayesh Alshukri, Abigail Bennett, Christopher P Cheyne, Antonio Eleuteri, Christopher Grierson, Mehrdad Mobayen-Rahni, Christopher Sampson, David Szmyt, Clare Thetford, Amu Wang; Helen Cooper, John Collins, Sue Howlin, John Kelly, Nathalie Massat, Gideon Smith, Vineeth Kumar, Chris A Rogers, Julia West, Naveed Younis
